# The mechanism effects of root exudate on microbial community of rhizosphere soil of tree, shrub, and grass in forest ecosystem under N deposition

**DOI:** 10.1038/s43705-023-00322-9

**Published:** 2023-11-20

**Authors:** Hang Jing, Huiling Wang, Guoliang Wang, Guobin Liu, Yi Cheng

**Affiliations:** 1 School of Geography, Nanjing Normal University, 210023 Nanjing, China; 2 State Key Laboratory of Soil Erosion and Dryland Farming on the Loess Plateau, Institute of Soil and Water Conservation, Northwest A&F University, 712100 Yangling, China; 3 Institute of Soil and Water Conservation, Chinese Academy of Science and Ministry of Water Resources, 712100 Yangling, China

**Keywords:** Microbial ecology, Plant ecology, Forest ecology

## Abstract

Forests are composed of various plant species, and rhizosphere soil microbes are driven by root exudates. However, the interplay between root exudates, microbial communities in the rhizosphere soil of canopy trees, understory shrubs, grasses, and their responses to nitrogen (N) deposition remains unclear. *Pinus tabulaeformis*, *Rosa xanthina*, and *Carex lancifolia* were used to investigate root exudates, rhizosphere soil microbial communities, and their responses to N application in forest ecosystem. Root exudate abundances of *P. tabulaeformis* were significantly higher than that of *R. xanthina* and *C. lancifolia*, with carbohydrates dominating *P. tabulaeformis* and *R. xanthina* root exudates, fatty acids prevailing in *C. lancifolia* root exudates. Following N application, root exudate abundances of *P. tabulaeformis* and *R. xanthina* initially increased before decreasing, whereas those of *C. lancifolia* decreased. Microbial number of rhizosphere soil of *C. lancifolia* was higher than that of *P. tabulaeformis* and *R. xanthina*, but there was insignificant variation of rhizosphere soil microbial diversity among plant species. N application exerted promotional and inhibitory impacts on bacterial and fungal numbers, respectively, while bacterial and fungal diversities were increased by N application. Overall, N application had negative effects on root exudates of *P. tabulaeformis*, inhibiting rhizosphere soil microbial populations. N application suppressed rhizosphere soil microbial populations by increasing root exudates of *R. xanthina*. Conversely, N application elevated nutrient content in the rhizosphere soil of *C. lancifolia*, reducing root exudates and minimally promoting microbial populations. This study highlights the importance of understory vegetation in shaping soil microbial communities within forests under N deposition.

## Introduction

Trees, shrubs, and grasses constitute the primary vegetation in forests, with different ecological niches but contributing equally importantly to the ecosystem. Yuan et al. [1] demonstrated that the priming effect in the rhizosphere soil of understory grasses was similar to that of canopy trees in subtropical forests. Although understory vegetation plays a critical role in forest soil material cycling [2], further verification is needed to understand the patterns of rhizosphere soil microbial communities between canopy trees and understory vegetations. Compared to trees, grasses and shrubs allocate a higher proportion of photosynthates underground to produce root exudates, which helps increase soil organic carbon content and microbial activity [3]. Therefore, soil respiration of grassland is 20% faster than that of forestland on the global scale [4]. Root exudates play a pivotal role in influencing rhizosphere soil microbes; their production directly impacts microbial activity, and their metabolites regulate microbial composition [5]. For example, legume roots secrete strigolactone to promote arbuscular mycorrhizal fungi, which fix more soil N and alleviate plant nutrient limitations [6]. It is evident that plants secrete specific root exudates to create a favorable rhizosphere environment, leading to apparent species-specific traits in rhizosphere soil microbial communities [7]. Grasses allocate a higher proportion of photosynthates to root exudates compared to trees, resulting in different root exudate compositions. These lead to faster soil biochemical reactions in grasslands than in forestlands and contribute to a species-specific microbial community. However, despite sharing the same forest ecosystem, it remains to be determined whether canopy trees, understory shrubs, and grasses exhibit species-specific traits in their root exudates and rhizosphere soil microbial communities.

In larch forests, N deposition altered the soil microbial communities and increased microbial biomass [8], whereas N deposition reduced soil biomass by 19.5% in red pine forests [9]. The responses of soil microbes to N deposition vary greatly among plant ecosystems, likely due to their different survival strategies and root exudates. Plants increase root exudates to enhance soil microbial activity and expedite material cycling when soil nutrient supply is insufficient, thus alleviating plant nutrient limitations [10]. However, N deposition directly alleviates plant nutrient limitations, leading to reduced carbon input of root exudates, and decreased soil microbial quantity and activity [11, 12]. Nevertheless, other studies have found that although N deposition alleviates plant nutrient limitations, root exudates and microbial activity were enhanced [13, 14]. This outcome may be attributed to the accelerated passive transport of exudates, due to increased nutrient content in plant roots. It is evident that diverse exudate yield strategies play an important role in the responses of soil microbes to N deposition, and different metabolites contribute to the specific soil microbial characteristics. Microbial community of rhizosphere soil of maize was apparently changed by N application through increasing the yield of phenols in root exudates [15]. Meanwhile, N application significantly altered the bacterial communities and increased the abundance of ammoxic bacteria in the rhizosphere soil of *Kandelia obovata*. This change is associated with the enhanced yield of amino acids and carbohydrates in root exudates [16]. N deposition influences rhizosphere soil microbial communities by changing the yield and composition of root exudates, and the specific mechanisms vary among plant species. Soil microbial communities of forestland and grassland respond differently to N deposition. Zhai et al. [17] demonstrated that soil respiration of *Metasequoia* forestland linearly increased with N additions in eastern China, while soil respiration of field grassland parabolically increased, with the fastest rate at medium N addition. Soil microbial community of grassland may be more susceptive to N application than that of forestland, and numerous studies exist regarding these ecosystems [18]. However, the responses of rhizosphere soil microbial communities of canopy trees, understory shrubs, and grasses to N deposition, and the driving mechanism of root exudates require clarification in forest ecosystems.

This study aims to test the following hypotheses: (1) Microbial quantity in the rhizosphere soil of understory grasses and shrubs is higher than that of canopy trees in forest ecosystems, and their microbial communities differ significantly, linked to species-specific root exudates. (2) N deposition affects rhizosphere soil microbial communities by changing root exudates, and the response of microbial communities in the rhizosphere soil of understory grasses is more susceptible than that of understory shrubs and canopy trees.

## Methods

### Study area description and experimental design

The study area is located at the Yichuan Field Experimental Station, Chinese Academy of Sciences, Shaanxi Province China, with geographical coordinates of 109°41'36”–110°32'44” E longitude and 35°42'39”–36°23'39” N latitude. The soil belongs to the gray forest soil (FAO soil classification). Pine trees are crucial species in forest ecosystems worldwide, and *P. tabulaeformis* is the most widely planted species in the study area. Its recent stand characteristics are as follows, average stand density, canopy density, diameter at breast height, tree height, forest volume, and leaf area index were 1400–1800 ha^−1^, 0.7, 10.0 cm, 11.2 m, 75.5 m^3^ hm^−2^, and 6.34, respectively. The understory vegetation of *P. tabulaeformis* forest mainly includes *R. xanthina*, *C. lancifolia*, *Lonicera japonica*, *Spiraea salicifolia*, *Ostryopsis davidiana*, *Elaeagnus pungens*, *Viburnum dilatatum*, and *Armeniaca sibirica*.

An N application experiment was conducted in the sampling field of *P. tabulaeformis* forest since 2014, with four N application treatments (0, 3, 6, 9 g N m^−2^ y^−1^ corresponding to N0, N3, N6, N9) based on the recent N deposition rate on the Loess Plateau of northwest China and the experimental designs from previous studies [18, 19]. Each N application treatment consisted of four replicate plots (each plot 10 m × 10 m) with similar terrain conditions. NH_4_NO_3_ was used as the N source, dissolved, and evenly sprayed on the soil surface before rainfall in April, June, August, and October annually. Based on the community composition, we selected *P. tabulaeformis* (canopy tree), *R. xanthina* (understory shrub), and *C. lancifolia* (understory grass) as the study subjects. Rhizosphere soils and exudates were sampled around fine roots with a diameter of < 0.5 mm, due to the fibrous root system of *C. lancifolia* and the consistent requirement among the three plant species.

### Soil sampling

Soil was sampled in October 2019 after 6 years of simulated N deposition. The in situ sampling method was employed to obtain rhizosphere soil samples, which is briefly explained as follows, a 30 cm depth of the soil profile (0–20 cm for sampling, 20–30 cm for standing) was excavated around specific plant species, and the root cut surfaces were exposed. A small spoon was used to collect rhizosphere soil from the 0–1 cm region around the fine roots of specific diameter classes. Non-rhizosphere soil was sampled from 0 to 20 cm depth in canopy gaps with no trees and sparse understory vegetation. A soil drill (5 cm in diameter, 20 cm in length) was used for the operation after removing litter and debris from the soil surface. After collection, soil samples were divided into two parts after removing foreign matter and passing through a 2 mm sieve. One part was stored at −80 °C for microbial diversity and quantity analyses, while the other part was used for chemical analysis after air-drying for several days to achieve a constant weight.

### Root exudate collection

Root exudates were collected using the modified static collection method proposed by Phillips et al. [20], which has been applied in many previous studies [21, 22]. Fine roots were carefully dug up, washed with distilled water, and wrapped in moist fabric tissue for 48 h to minimize damage. Exudates were collected from 3 to 5 fine roots with minimal damage, and fabric tissues were covered with litter to prevent light and water loss. After being washed and drained, the fine roots were incubated in a 50 mL centrifuge tube filled with carbon-free nutrient solution (0.5 mM NH_4_NO_3_, 0.1 mM KH_2_PO_4_, 0.2 mM K_2_SO_4_, 0.2 mM MgSO_4_, 0.3 mM CaCl_2_) and 750 μm diameter glass beads for 3 days. Carbon-free nutrient solution and glass beads were used to replicate the chemical and physical environment of rhizosphere soil. During the incubation, the centrifuge tube was covered with aluminum foil and gently capped to prevent environmental disturbances. After 3 days, the solution in the centrifuge tube was aspirated using a syringe, and the centrifuge tube and glass beads were rinsed twice more with carbon-free trap solution. After collection, these solutions were immediately transported back to the laboratory and passed through filter membranes at 4 °C as soon as possible. These exudate samples were frozen at −20 °C in the laboratory for metabolomics analysis, with six replicates per treatment.

### Microbial quantity and community analysis

Real-time PCR was used to evaluate the microbial quantity of bacteria and fungi. The detailed methods, procedures, and instrument settings can be found in the Supplementary method. Soil microbial communities were evaluated using 16S, ITS rRNA genes, with operations including DNA extraction, Illumina HiSeq2500 sequencing, and data processing. DNA samples were extracted from fresh soil samples (0.4 g) using a Soil DNA Kit (Omega Bio-tech, USA) following the manufacturer’s instructions. These samples were then stored at −80 °C in the laboratory before high-throughput sequencing on the Illumina MiSeq PE300 platform. The specific procedures and instrument parameters of sequencing were detailed in the Supplementary method. After sequencing, the sequences were quality-filtered, denoised, merged, and chimeras were removed, following the Quantitative Insights into Microbial Ecology (QIIME) workflow. Meanwhile, sequences with lengths < 50 bp and unresolved nucleotides were eliminated [23]. Thus, the remaining sequences were clustered into operational taxonomic units (OTUs) using USEARCH at a 98% similarity and 97% identity rate [24]. The bacterial 16S rRNA genes were annotated according to the SILVA database (https://www.arb-silva.de/) [25], while the fungal ITS genes were annotated according to the UNITE database (https://unite.ut.ee/). A phylogenetic tree was performed according to the “qiime phylogeny align-to-tree-mafft-fasttree” operation, aligning operational taxonomic units (OTUs) with mafft [26], and annotating sections lacking phylogenetic information. The tree file was then generated by the Fast Tree algorithm [27]. The OTU table was flattened using a sparse method before further data analysis.

### Non-targeted metabolomics analysis of root exudates

The composition and relative abundance of root exudates of the three plant species were analyzed through non-targeted metabolomics using gas chromatography-time-of-flight mass spectrometry (GC-MS). Key procedures included exudate extraction, machine detection and off-line data processing. Detailed operating procedures and instrument parameters are described in the Supplementary method.

### Soil chemical properties analysis

The content of soil organic carbon (SOC) was determined using the external heating of potassium dichromate method [28]; the content of soil N was determined through the Kjeldahl determination method [29]; the content of soil phosphorus (P) was determined using molybdenum fluorescence colorimetric method and the content of available phosphorus (AP) was determined through sodium bicarbonate extraction molybdenum antimony anti-colorimetric method [30]. The contents of ammonium (NH_4_^+^) and nitrate (NO_3_^−^) were determined using the continuous flow analyzer method. The pH value was measured using an automatic titrator method.

### Data analysis and mapping

The shannon-wiener, chao1, observed species, and simpson indices were calculated at the OTU level to evaluate the α diversity of soil microbial communities. Besides, the effects of N application and plant species on the α diversity of these communities were identified using two-way ANOVA. Microbial quantities were visualized using bar charts, and differences among N applications and plant species were evaluated using the least significant difference (LSD) multiple comparison analysis. The normal distributions of α diversities and quantities of soil microbial communities were confirmed before two-way ANOVA and LSD analyses. The differences of soil microbial community structure were identified through non-metric multidimensional scaling (NMDS) using Bray-Curtis distance, and comparisons between any two treatments were evaluated using pairwise permutational analysis of variance (PERMANOVA). The aforementioned statistical analyses and visualizations were performed using R software (R Development Core Team 2018). Linear discriminant analysis effect size (LEfSe) was conducted on the Galaxy/Huttenhower platform (http://huttenhower.org/galaxy/) to identify differentially abundant microbial populations across treatments. This was done using a threshold of LDA > 2.0, and the results were visualized in a heatmap format. Principal coordinates analysis (PCoA) with Bray-Curtis distance was used to examine the effects of experimental treatments on root exudate compositions and for quality control of the operation process. The relative abundance of root primary metabolites was obtained by calculating the summation of the peak area of each metabolite by the summation of the peak area of all metabolites, and visualized using bar charts. NMDS and bar charts were generated using R software and its built-in functions. Partial least squares discriminant analysis (PLS-DA) was conducted using SIMCA 14.1 software (Umetrics, Umea, Sweden) to identify differential exudates across treatments, applying a threshold of variable influence on projection (VIP) values > 2.0 and *p* < 0.05. In addition, the differences root exudate composition were illustrated in a score scatter plot based on PLS-DA. The fit of the PLS-DA model was then verified through a permutation test.

SparCC co-occurrence network analysis is a method to propose relationships between differential microbial populations and functional metabolites. It involves calculating correlation coefficients and performing permutation tests to determine *p* values. The iteration parameter was set to the default-i 20, and 1000 bootstrap permutations were carried out. This analysis was conducted using the M2IA platform (http://m2ia.met-bioinformatics.cn/). Partial least squares path model (PLS-PM) was used to analyze the mechanism of N application on soil microbial communities by affecting soil chemical properties and root exudates among plant species. N application rates were reported as 0, 3, 6, 9 g N m^−2^ y^−1^, while soil chemical properties included those that exhibited significant changes under N application (ANOVA, *p* < 0.05). Microbial community and root exudate were in modules that implied the first principal coordinates (PCo1) of differential microbial populations and metabolites among treatments generated by Bray-Curtis distance matrices. Path coefficients were present in the modules and calculated after 1000 bootstraps. The model’s fit was assessed using the index of Goodness of Fit (GOF). The analysis was conducted using the “plspm” package of R software.

## Results

### Microbial quantity, richness, and composition of rhizosphere soils

**Fig. 1:**
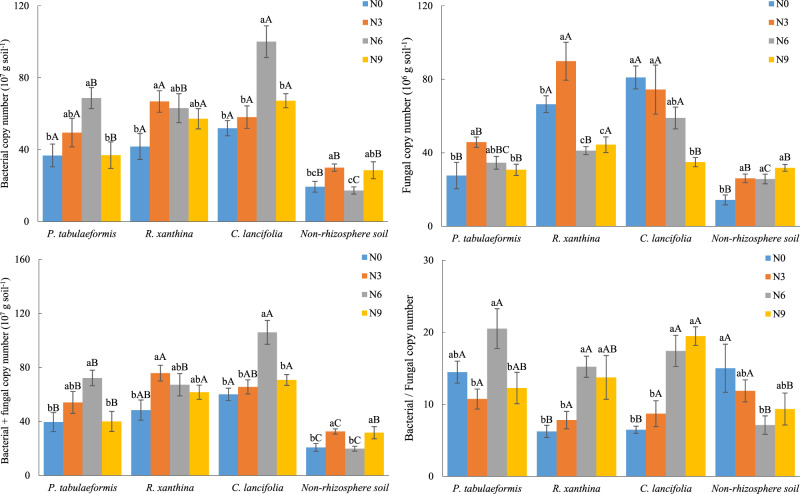
Effects of N application on microbial quantity of rhizosphere soil of different plant species and non-rhizosphere soil. Vertical bars indicate the standard errors of means (*n* = 4). Values followed by different lowercase letters indicate significant differences among the N applications, values followed by different uppercase letters indicate significant differences among plant species and non-rhizosphere soil, (*p* < 0.05). N0, N3, N6, N9 are 0, 3, 6, and 9 g N m^−2^ y^−1^, respectively.

There was a significant difference in microbial copy numbers of rhizosphere soils among plant species (*p* < 0.05, Fig. 1). Specifically, the highest total microbial and bacterial copy numbers were obtained in the rhizosphere soil of *C. lancifolia*, followed by the rhizosphere soils of *R. xanthina* and *P. tabulaeformis*. In contrast, the highest fungal copy numbers were observed in the rhizosphere soils of *C. lancifolia* and *R. xanthina*, whereas the lowest fungal copy number was found in the rhizosphere soil of *P. tabulaeformis*. Moreover, the impact of N application on microbial copy numbers of rhizosphere soil varied among plant species. In detail, the fungal copy number of *C. lancifolia* rhizosphere soil was inhibited by N application, whereas the bacterial and total microbial copy numbers exhibited a parabolic response, with the largest value obtained in the N6 treatment. In *P. tabulaeformis* rhizosphere soil, microbial total copy number showed a parabolic response to N application, with the bacterial copy number peaking in the N6 treatment and the fungal copy number peaking in the N3 treatment. The bacterial and fungal copy numbers in the rhizosphere soil of *R. xanthina* initially added and then decreased with N applications, with the largest value obtained in the N3 treatment.

Microbial diversity indices of non-rhizosphere soil, including chao1, simpson, shannon, and observed species were significantly higher than those of rhizosphere soils (*p* < 0.05, Table 1). However, the microbial diversity indices of rhizosphere soils showed no significant differences among plant species. N application significantly increased bacterial diversity indices, with the largest values obtained in the N6 or N9 treatments. Fungal diversity indices showed a nonlinear response to N application, with the largest values obtained in the N6 treatment.

**Table 1:** Richness and diversity measures of microbial community in rhizosphere soil of different plant species and non-rhizosphere soil among N applications (mean ± SE, *n* = 4).

Species	Treatment	Fungi				Bacteria			
		Chao1	Simpson	Shannon	Observed species	Chao1	Simpson	Shannon	Observed species
*P. tabulaeformis*	N0	213.3 ± 26.8	0.57 ± 0.14	3.05 ± 0.91	204.4 ± 27.4	1760.5 ± 240.2	0.998 ± 0.0005	9.63 ± 0.20	1486.4 ± 172.7
	N3	214.3 ± 32. 2	0.68 ± 0.02	3.09 ± 0.15	206.9 ± 28.8	1574.1 ± 123.8	0.998 ± 0.0003	9.56 ± 0.11	1373.3 ± 88.7
	N6	239.2 ± 49.1	0.92 ± 0.02	5.04 ± 0.43	235.4 ± 48.0	1763.6 ± 74.6	0.998 ± 0.00008	9.70 ± 0.02	1495.4 ± 43.2
	N9	172.1 ± 11.5	0.79 ± 0.05	3.74 ± 0.29	171.0 ± 11.6	2005.3 ± 95.0	0.998 ± 0.00008	9.86 ± 0.06	1680.7 ± 70.1
*R. xanthina*	N0	222.2 ± 33.3	0.78 ± 0.15	4.20 ± 0.89	214.0 ± 33.5	1591.1 ± 97. 5	0.998 ± 0.0002	9.57 ± 0.11	1364.0 ± 74.9
	N3	171.8 ± 21.2	0.68 ± 0.13	3.22 ± 0.69	165.3 ± 21.3	1876.6 ± 212.2	0.996 ± 0.002	9.65 ± 0.25	1599.7 ± 169.6
	N6	294.1 ± 61.9	0.89 ± 0.04	4.87 ± 0.45	287.6 ± 61.5	1692.3 ± 126.9	0.998 ± 0.0002	9.61 ± 0.11	1427.7 ± 99.0
	N9	213.3 ± 34.8	0.82 ± 0.09	4.30 ± 0.62	213.0 ± 34.8	1865.6 ± 43.9	0.998 ± 0.0002	9.78 ± 0.05	1585.0 ± 29.7
*C. lancifolia*	N0	226.0 ± 15.6	0.77 ± 0.05	3.58 ± 0.29	217.2 ± 14.9	1566.0 ± 67.8	0.998 ± 0.0001	9.62 ± 0.07	1384.9 ± 56.7
	N3	202.3 ± 20.4	0.86 ± 0.054	4.32 ± 0.53	200.7 ± 20.0	1663.4 ± 176.7	0.998 ± 0.0002	9.63 ± 0.11	1441. 5 ± 126.2
	N6	237.1 ± 27.1	0.84 ± 0.03	4.35 ± 0.36	234.5 ± 27.0	1639.8 ± 63.4	0.995 ± 0.002	9.58 ± 0.15	1462.9 ± 52.4
	N9	198.8 ± 16.7	0.80 ± 0.11	4.37 ± 0.75	195.2 ± 17.6	1821.0 ± 75.3	0.998 ± 0.0002	9.81 ± 0.07	1592.1 ± 62.4
Non-rhizosphere soil	N0	227.9 ± 21.7	0.92 ± 0.05	5.75 ± 0.59	227.4 ± 21.7	1901.3 ± 82.6	0.997 ± 0.0005	9.72 ± 0.09	1617.6 ± 59.9
	N3	294.8 ± 31.3	0.97 ± 0.01	6.39 ± 0.23	291.3 ± 28.5	2557.5 ± 311.0	0.998 ± 0.0001	10.17 ± 0.17	2065.1 ± 213.7
	N6	292.1 ± 17.9	0.98 ± 0.01	6.75 ± 0.17	291.9 ± 17.91	2762.8 ± 70.5	0.998 ± 0.0002	10.23 ± 0.07	2225.1 ± 54.0
	N9	243.8 ± 13.6	0.97 ± 0.01	6.46 ± 0.10	243.6 ± 13.5	2265.6 ± 272.9	0.998 ± 0.0002	10.00 ± 0.15	1871.6 ± 200.6
Factors (df)									
N application (3)	*F*	2.84*	2.79*	3.64*	2.94*	2.8*	0.51	2.19	2.94*
Species (3)		2.68*	6.13**	19.92**	3.2*	17.03***	0.91	8.40***	15.94***
Interaction (6)		0.83	0.94	0.76	0.79	1.85	1.16	1.07	1.74

The data represent at the OTU level. N0, N3, N6, N9 are 0, 3, 6, and 9 g N m^–2^ y^–1^, respectively.

***(*p*  <  0.001), **(*p*  <  0.01), and *(*p*  < 0.05) indicate significant differences among the treatments based on a two-way ANOVA test.

**Fig. 2:**
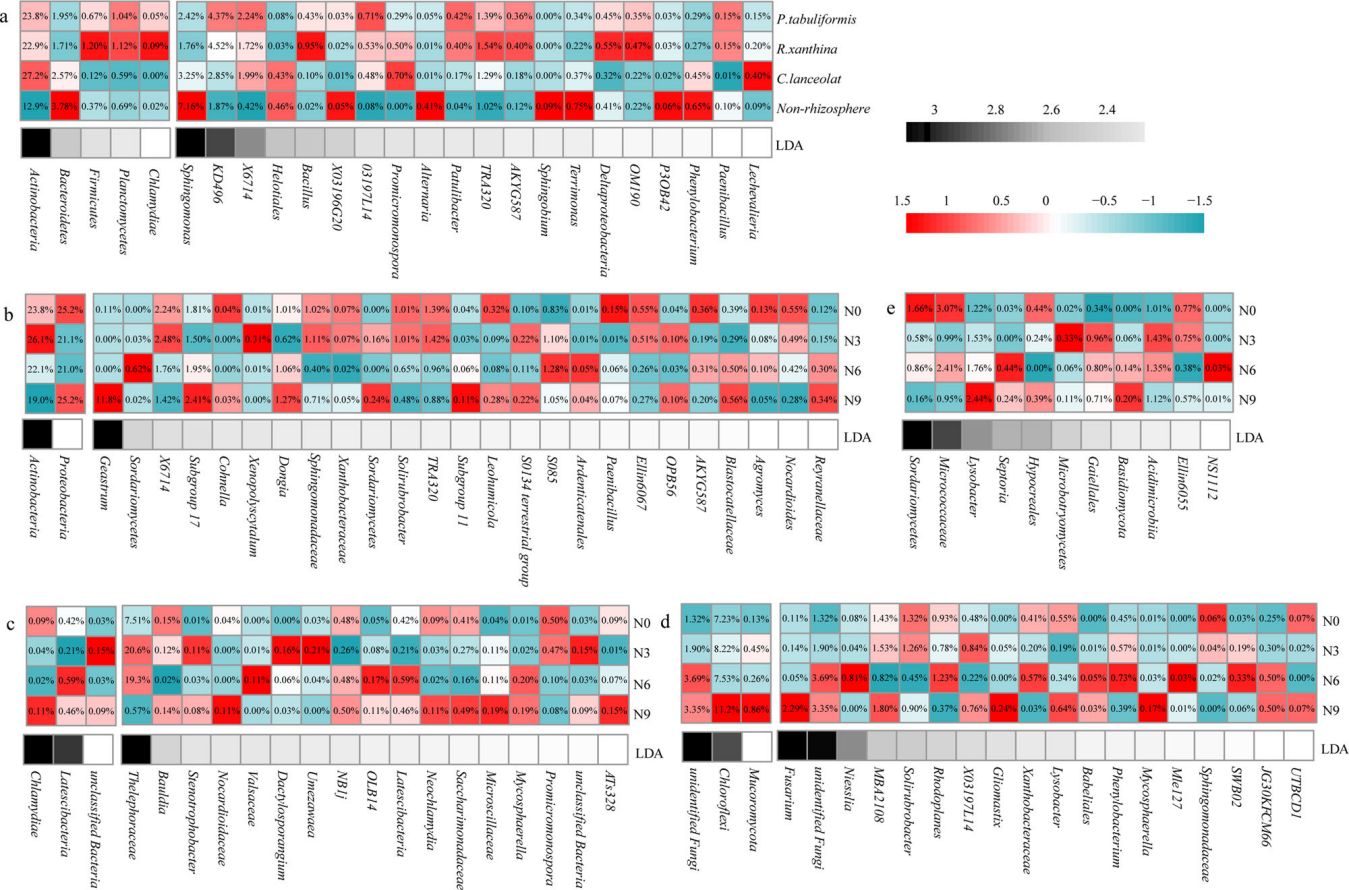
Heatmap of LEfSe of rhizosphere soil microbial populations across the different plant species at N0 treatment and across the N application treatments at different plant species (*n* = 4). Relative abundance of microbial populations are shown in boxes, red indicates the greater relative abundance after normalization among treatments, blue indicates the lower relative abundance after normalization among treatments. Black indicates the larger LDA value than 2. **a** Microbial populations across the plant species at N0 treatment; **b** Microbial populations across the N application treatments in the rhizosphere soil of *P. tabulaeformis*; **c** Microbial populations across the N application treatments in the rhizosphere soil of *R. xanthina*; **d** Microbial populations across the N application treatments in the rhizosphere soil of *C. lancifolia*; **e** Microbial populations across the N application treatments at non-rhizosphere soil. N0, N3, N6, N9 are 0, 3, 6, and 9 g N m^−2^ y^−1^, respectively.

Significant differences were found in microbial communities among non-rhizosphere and rhizosphere soils (*p* < 0.05, Table 2 and Supplementary Fig. S1). LDA revealed that 9 microbial populations were dominant in non-rhizosphere soil (Fig. 2a); 5 microbial populations were dominant in the rhizosphere soil of *C. lancifolia*; 9 microbial populations were dominant in the rhizosphere soil of *R. xanthina*; 5 microbial populations were dominant in the rhizosphere soil of *P. tabulaeformis*. N application significantly changed the soil microbial community (*p* < 0.05, Table 2), with the greatest changes observed between N3 and N9 treatments. Moreover, there was an insignificant interaction effect between N application and plant species, indicating that soil microbial community structure of non-rhizosphere and rhizosphere soils had similar responses to N application. However, the differential microbial populations responding to N application varied among non-rhizosphere soil and rhizosphere soils. LDA showed that 11, 27, 20, and 21 differential microbial populations were generated in non-rhizosphere soil and rhizosphere soils of *P. tabulaeformis*, *R. xanthina*, and *C. lancifolia* after N application, respectively (Fig. 2b–e).

**Table 2:** Results of PERMANOVA of Bray-Curtis distances testing the effect of N application and plant species on soil microbial communities.

Global tests	Fungi			Bacteria		
	Pseudo-F	*R*^2^	*p* value	Pseudo-F	*R*^2^	*p* value
N application	1.62	0.07	0.001**	1.53	0.06	0.003**
Plant species	3.21	0.13	0.001**	4.98	0.19	0.001**
N application × Plant species	0.99	0.13	0.526	1.12	0.13	0.064

Pairwise tests	Fungi			Bacteria		
	*t*-statistic	*R*^2^	*p* value	*t*-statistic	*R*^2^	*p* value
N0 vs. N3	0.97	0.03	0.443	1.01	0.03	0.365
N0 vs. N6	1.42	0.05	0.055	1.15	0.04	0.194
N0 vs. N9	1.61	0.05	0.022*	1.41	0.04	0.058
N3 vs. N6	1.53	0.05	0.022*	1.30	0.04	0.089
N3 vs. N9	1.75	0.06	0.007**	1.41	0.04	0.041*
N6 vs. N9	1.47	0.05	0.01*	1.28	0.04	0.108
*P. tabulaeformis* vs. *R. xanthina*	1.47	0.05	0.041*	1.33	0.04	0.02*
*P. tabulaeformis* vs. *C. lancifolia*	1.92	0.06	0.009**	3.27	0.10	0.001**
*P. tabulaeformis* vs. Non-rhizosphere soil	5.47	0.15	0.001**	6.79	0.18	0.001**
*R. xanthina* vs. *C. lancifolia*	1.47	0.05	0.025*	3.14	0.09	0.001**
*R. xanthina* vs. Non-rhizosphere soil	4.68	0.13	0.001**	6.84	0.19	0.001**
*C. lancifolia* vs. Non-rhizosphere soil	4.67	0.13	0.001**	7.77	0.21	0.001**

N0, N3, N6, N9 are 0, 3, 6, and 9 g N m^–2^ y^–1^, respectively.

**(*p* <  0.01) and *(*p*  < 0.05) indicate significant differences among the treatments.

### Root exudates of different plant species

**Fig. 3:**
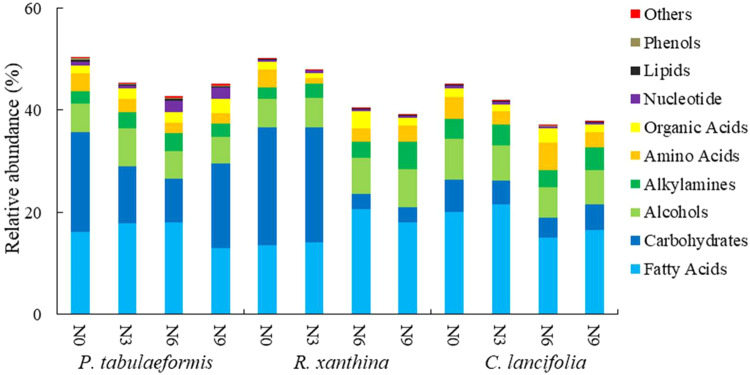
Relative abundance of the root primary metabolites among N application treatments and plant species. The relative abundance is calculated by the summation of the peak area of each metabolite by the summation of the peak area of all metabolites. Primary metabolites with an average relative abundance of < 0.1% are classified as others. N0, N3, N6, N9 are 0, 3, 6, and 9 g N m^−2^ y^−1^, respectively.

**Fig. 4:**
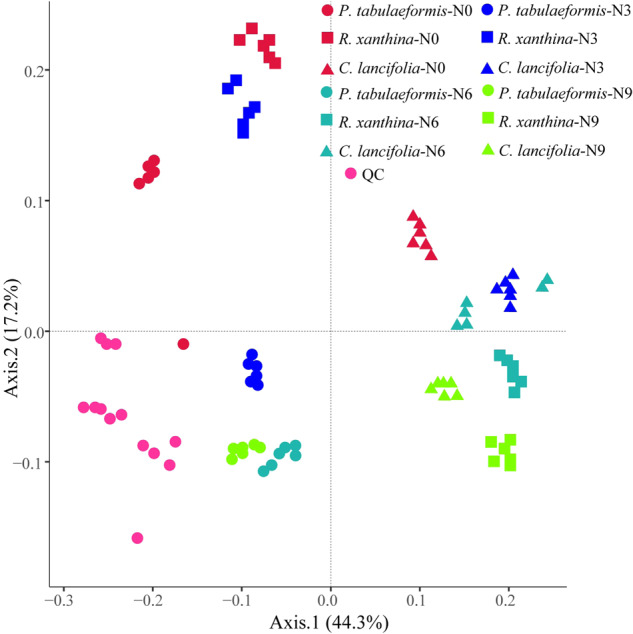
PCoA based on Bray-Curtis distances depicting the root exudate profiles among N application treatments and plant species. N0, N3, N6, N9 are 0, 3, 6, and 9 g N m^−2^ y^−1^, respectively. QC quality control samples.

**Fig. 5:**
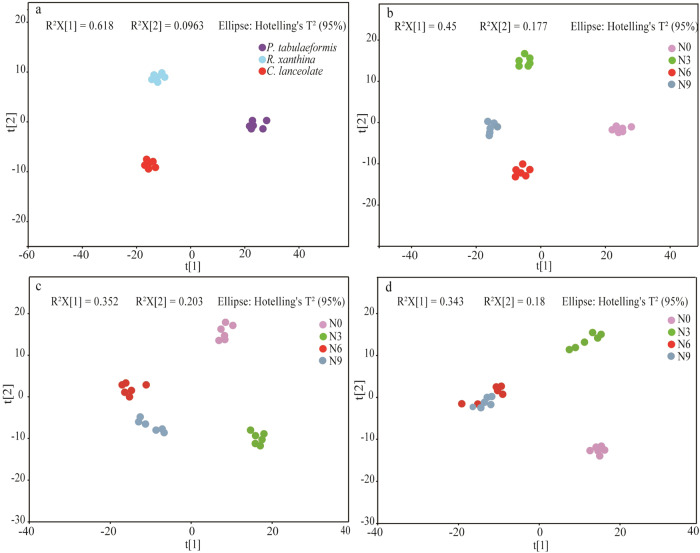
Score scatter plot of PLS-DA for identified differential root exudates across the plant species at N0 treatment and across the N application treatments at different plant species (*n* = 6). **a** Root exudate profiles across the plant species at N0 treatment; **b** Root exudate profiles of *P. tabulaeformis* across the N application treatments; **c** Root exudate profiles of *R. xanthina* across the N application treatments; **d** Root exudate profiles of *C. lancifolia* across the N application treatments. N0, N3, N6, N9 are 0, 3, 6, and 9 g N m^−2^ y^−1^, respectively.

**Fig. 6:**
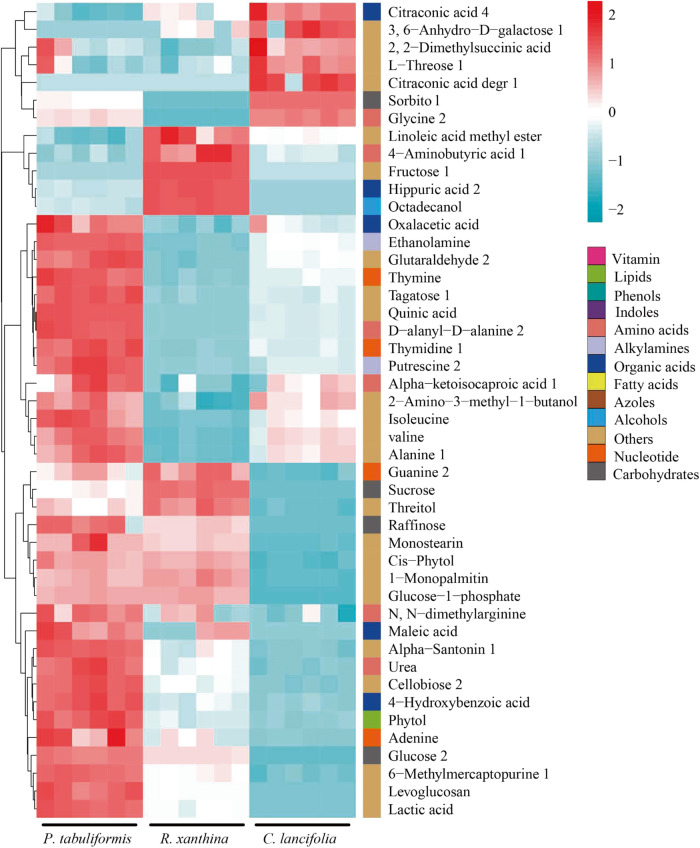
Clustered heatmap of the differential root exudates and their associated primary metabolites among plant species (VIP > 2.0 and *p* < 0.05).

*C. lancifolia* root exudates exhibited a relatively high abundance of fatty acids, whereas root exudates of *P. tabulaeformis* and *R. xanthina* showed relatively high abundances of carbohydrates (Fig. 3). The patterns of root exudates among plant species were significantly different (Figs. 4, 5). In general, the relative abundance of root exudates of *P. tabulaeformis* was the highest, followed by *R. xanthina* and *C. lancifolia* (Fig. 6). A total of 46 differential metabolites were identified among plant species, with 34 metabolites having the highest relative abundances in the root exudate of *P. tabulaeformis*, 5 metabolites having higher relative abundances in the root exudate of *R. xanthina*, and 7 metabolites having higher relative abundances in the root exudate of *C. lancifolia*.

Root exudates of *P. tabulaeformis* showed the largest difference among N application treatments, while root exudates of *R. xanthina* and *C. lancifolia* were similar in the N6 and N9 treatments, but significantly different in other N application treatments (Fig. 5). Specifically, N application significantly altered the relative abundances of 87 metabolites of *P. tabulaeformis* (Fig. S2a), with most metabolites being dominant in the N0 and N6 treatments. A total of 101 metabolites of *R. xanthina* showed significant responses to N application, with most metabolites having the higher relative abundances in the N3 treatment (Fig. S2b). Furthermore, after N application, the relative abundances of 105 metabolites of *C. lancifolia* significantly changed, and the relative abundances of dominant metabolites gradually decreased with N applications (Fig. S2c). Across all kinds of primary metabolites, organic acids, amino acids, fatty acids, carbohydrates, and nucleotides were the main metabolites in response to N application.

### N application altered the microbial community by changing root exudates and chemical properties of rhizosphere soil

**Fig. 7:**
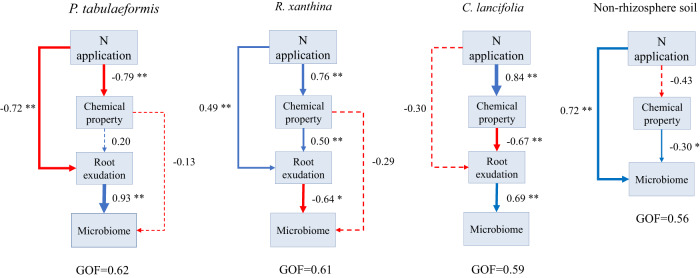
Path analysis of the effects of N application on root exudate, soil chemical property, and microbial community in rhizosphere soil of different plant species and non-rhizosphere soil. The width of the arrow is proportional to the absolute value of path coefficient. Red indicates negative effects, and blue indicates positive effects. coefficient is calculated after 1000 bootstraps. Solid lines indicate significant effects between variables, while dashed lines indicate insignificant effects between variables. GOF, goodness of fit. *p *<* 0.05; **p < 0.01.

In general, N application inhibited the root exudate abundances of *P. tabulaeformis*, which had a negative effect on the microbial community of rhizosphere soil (Fig. 7). In the rhizosphere soil of *R. xanthina*, N application inhibited the microbial populations by increasing the abundance of root exudates. Moreover, N application also affected the microbial community of rhizosphere soil of *R. xanthina* by changing the root exudate abundances and soil properties. Besides, N application reduced the root exudate abundances by increasing the soil nutrient contents, which is not conducive to increasing the microbial populations of rhizosphere soil of *C. lancifolia*. In addition, N application directly promoted the microbial community of non-rhizosphere soil.

**Fig. 8:**
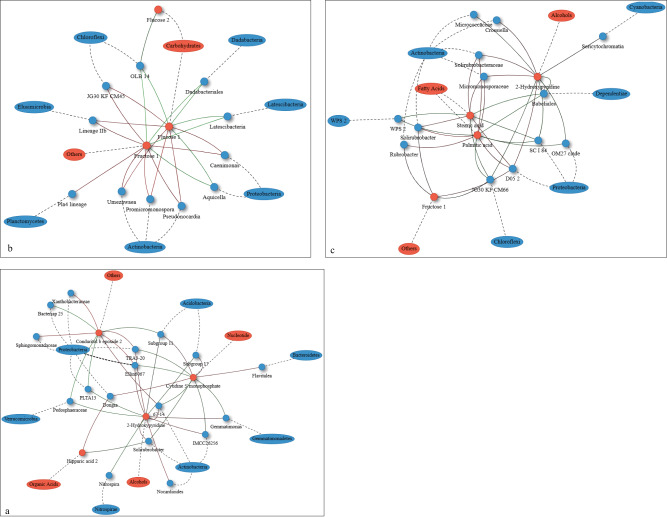
Network of correlation analysis between differential exudates and bacterial populations across N application treatments. Only significant correlations are shown (*p* < 0.05). The green lines show positive correlations, the red lines show negative correlations, and the dashed lines show the relationship between secondary and primary metabolites, and the relationship between genus and phylum. **a** Correlation analysis between root exudates and bacterial populations of *P. tabulaeformis*; **b** Correlation analysis between root exudates and bacterial populations of *R. xanthina*; **c** Correlation analysis between exudates and bacterial populations of *C. lancifolia*.

In the rhizosphere soil of *P. tabulaeformis*, root-produced Conduritol b epoxide 2, Cytidine 5´ monophosphate, and 2-Hydroxypyridine were significantly correlated with the populations of *Proteobacteria*, *Actinobacteria*, and *Acidobacteria*, with 16 positive correlations and 16 negative correlations (*p* < 0.05, Fig. 8a). Fructose 1 and Glucose 1 produced by the roots of *R. xanthina* were significantly correlated with the populations of *Actinobacteria*, *Proteobacteria*, and *Chloroflexi*, with 8 positive correlations and 14 negative correlations (*p* < 0.05, Fig. 8b). In the rhizosphere soil of *C. lancifolia*, root-produced Palmitic acid, 2-Hydroxypyridine, and Stearic acid were significantly correlated with the populations of *Actinobacteria* and *Proteobacteria*, with 16 positive correlations and 16 negative correlations (*p* < 0.05, Fig. 8c). Furthermore, there were significant positive correlations between Glucose 1, Fructose 1, and Stearic acid produced by *P. tabulaeformis* and the fungal populations of *Ascomycota*, *Basidiomycota*, and *Mortierellomycota* (*p* < 0.05, Fig. S3a). Root-produced 2-Hydroxypyridine and Conduritol b epoxide 2 by *R. xanthina* were significantly positively correlated with populations of *Basidiomycota* (*p* < 0.05, Fig. S3b). Palmitic acid, Stearic acid, and 2-Hydroxypyridine produced by the roots of *C. lancifolia* showed significant positive correlations with the populations of *Ascomycota* (*p* < 0.05, Fig. S3c).

## Discussion

### Root exudates among plant species and N application effects

The relative abundance of root exudates of *P. tabulaeformis* was higher than that of *R. xanthina* and *C. lancifolia*, which contradicted our hypothesis. In wetland ecosystems, root exudate yield of *Lythrum salicari* was the highest, followed by the *Zizania caduciflora* and *Canna indica* [31]. These consequences were relevant to vegetation biomass, *Lythrum salicari* has an actively growing root system with rich nutrient content, leading to a high yield of root exudates. In the present study, the high abundance of root exudates of *P. tabulaeformis* (tree) may be related to its rich root biomass and developed root system. There were 46 differential metabolites among plant species, and most of them were dominant in the root exudates of *P. tabulaeformis*, supporting the difference in root exudate yield among plant species in *P. tabulaeformis* forest. Carbohydrates were relatively abundant in the root exudates of *P. tabulaeformis* and *R. xanthina*, while fatty acids had a relatively high abundance in the root exudates of *C. lancifolia*. Different kinds of metabolites have varied functions. Fatty acids have the ability to strengthen plant resistance and inhibit pathogenic bacteria [32, 33]. Carbohydrates provide energy for microbes mainly through passive transport, and the yield rate is proportional to the carbohydrate content of roots. The relatively high abundance of fatty acids indicates that roots can resist more soil diseases and foster a beneficial rhizosphere microbial community of *C. lancifolia*. The relatively high abundance of carbohydrates in the root exudates of *P. tabulaeformis* and *R. xanthina* may be related to the high nutrient contents of their root systems, which could promote the quantity and diversity of rhizosphere soil microbes.

*P. tabulaeformis* root exudates were more susceptible to N application than *R. xanthina* and *C. lancifolia* (Fig. 5). These results are inconsistent with our hypothesis. The responses of root exudates to environmental changes varied among plant species, driven by their homeostasis and survival strategies [10–14]. For instance, *Balanites aegyptiaca* and *Acacia raddiana* were drought-toletant species that promoted *Azospirillum brasilense* reproduction by increasing galactan protein yield in root exudates [34]. This case was beneficial for root growth, thus alleviating drought stress. In contrast, *Tamarindus indica* root exudates did not change with soil moisture, providing no advantage for *Azospirillum brasilense* populations or drought resistance. In this study, the different responses of root exudates to N application may be related to their diverse homeostasis and survival strategies. Low N application alleviated the limitations of *P. tabulaeformis* and *R. xanthina*, and promoted root nutrient contents and exudate abundances. However, when nutrient requirements are met through high N application [35], root physiological activity and exudate yield may decrease. Following N application, *C. lancifolia* root exudates were significantly inhibited due to its low nutrient requirement, indicating marked differences in its homeostasis compared to *P. tabulaeformis* and *R. xanthina*. Furthermore, non-targeted metabolomics analysis demonstrated that metabolites responding to N application in *P. tabulaeformis* forests mainly belonged to organic acids and amino acids, which was consistent with studies on Alfalfa and Arabidopsis root exudates using similar methods [36, 37]. Organic acids alter soil pH, increase soil nutrient content, and play a crucial role in establishing beneficial microbial communities, providing substrates for biochemical reactions [38, 39]. Amino acids not only supply available N for soil microbes, but can also be reabsorbed by plant roots [40, 41].

### Rhizosphere soil microbial communities among plant species and N application effects

The microbial copy number of rhizosphere soil was apparently higher than that of non-rhizosphere soil, which is similar to other studies [42]. The microbial diversity of rhizosphere soil was lower compared to that of non-rhizosphere soil, indicating that rhizosphere environment is favorable for some microbes while being unfavorable for others, resulting in microbial diversity reduction. In this study, the microbial copy number of rhizosphere soil of *C. lancifolia* was apparently higher compared to that of *R. xanthina* and *P. tabulaeformis*, supporting our hypothesis. The microbial biomass of rhizosphere soil of *Artemisia ordosica* was found to be higher than that of rhizosphere soil of *Caragana intermedia* in a grassland ecosystem [43]. This result was attributed to the high content of total N, available potassium, AP, and neutral pH in the rhizosphere soil of *Artemisia ordosica*. The turnover of roots, quantity, and quality of litters were found to be the main factors affecting microbial biomass in the rhizosphere soils of *Secale cereale, Agropyron repens*, and *Cirsium arvense* [44]. In this study, the rapid decomposition of root litter and the rich soil nutrient might be the reasons for the higher microbial quantity in the rhizosphere soil of *C. lancifolia*.

Interestingly, the changes in root exudate abundance and microbial quantity among plant species were found to be inversely related, which deviated from previous studies [15, 16]. Root exudates are generally considered a crucial factor in promoting microbial reproduction, leading to a positive correlation between root exudate abundance and rhizosphere soil microbial quantity. However, some researches have suggested that the role of root exudates may be overestimated due to their low yield rate, while the high yield of rhizodeposits (litters and dead tissues) is a key factor in determining rhizosphere microbial reproduction [45, 46]. Consequently, rhizosphere microbial quantity is influenced by various factors such as root exudates, rhizodeposits, and soil chemical properties, resulting in significant differences among plant species. In this study, no significant difference in rhizosphere soil microbial diversity was observed among plant species. Soil (rhizosphere) microbial quantity is more sensitive to environmental changes compared to diversity, which is related to the fact that microbial reproduction is more influenced by nutrient content, while microbial species are less affected [47, 48]. Furthermore, the microbial compositions of rhizosphere soil varied among plant species [49]. *Bacteroidetes* and *Sphingomonas* were predominant in non-rhizosphere soil, as they were common soil microbial populations [50, 51]. The relatively high abundances of *Actinobacteria* and *Lechevalieria* in the rhizosphere soil of *C. lancifolia* may be attributed to their low nutrient requirements and slow yield of root exudates [52]. *Firmicutes*, *Bacillus*, etc., were dominant in the rhizosphere soil of *R. xanthina*, while *Patulibacter*, *Paenibacillus*, etc., were dominant in the rhizosphere soil of *P. tabulaeformis*. These microbial populations possess the ability to use polysaccharide and macromolecular substrate, with their high abundances being linked to the rapid yield of root exudates [53, 54].

Following N application, the fungal copy number of rhizosphere soil of *C. lancifolia* experienced a greater decrease than that of *R. xanthina* and *P. tabulaeformis*, aligning with our hypothesis. A global-scale meta-analysis revealed that N application reduced microbial respiration and biomass by 8% and 20%, respectively, and these inhibitory effects were associated with soil acidification and nutrient imbalance [55]. However, a study on *Larix principis-rupprechtii* plantations demonstrated that soil acidification was insignificant after N application, while nutrient limitations for plants and soil microbes were alleviated, thereby accelerating microbial reproduction [8]. The responses of microbial populations to N application varied greatly due to different plant nutrient requirements and soil conditions. Fungal reproduction was inhibited due to changes in soil nutrient imbalance [56], with fungal reproduction of rhizosphere soil of *C. lancifolia* being more responsive to N application than that of *R. xanthina* and *P. tabulaeformis*. This result might be attributed to grasses being more susceptible to environmental changes (e.g., litter decomposition and root exudate secrete) and having lower demands for soil nutrients. Similarly, after N application, bacterial reproduction of rhizosphere soil of *C. lancifolia* changed more noticeably than that of *R. xanthina* and *P. tabulaeformis*. The differences showed that N application significantly promoted bacterial reproduction, with the largest copy numbers obtained in the N6 or N9 treatments. Bacterial and fungal reproductions respond differently to environmental changes, as confirmed in previous studies [57, 58]. Our results corroborated these distinct changes in fungal and bacterial populations and found that N requirements of bacterial reproduction were higher than those of fungal reproduction.

Regarding soil microbial diversity, N application enhanced both bacteria and fungi, with the rhizosphere soil of different plant species experiencing similar changes. Diversity represents the richness of soil microbial populations, and increased diversity indicates that N application benefits the environment for most microbes, primarily bacteria [59]. The similar changes of microbial diversity of rhizosphere soils among plant species after N application may be related to the different sensitivities of microbial diversity and quantity to environmental changes [60]. Besides, the dominant microbial populations of rhizosphere of *C. lancifolia* and *R. xanthina* changed more significantly than those of rhizosphere of *P. tabulaeformis* following N application. These findings are supported by the results of soil microbial quantity, further confirming that the microbial community of rhizosphere soils of *C. lancifolia* and *R. xanthina* is more susceptible to N application than that of *P. tabulaeformis*.

### The mechanisms of N application affect microbial community by altering root exudate and chemical property of rhizosphere soil

Microbial communities of rhizosphere soil of different plant species exhibited varied mechanisms in response to N application, which was consistent with our hypothesis and other studies [16, 17]. Root exudates played a crucial role when N application substantially increased fungal diversity and dominant populations of rhizosphere soil of *Bothriochloa ischaemum* [61]. This mechanism was related to the high yield of long-chain organic acids in root exudates. In contrast, rhizosphere soil chemical properties were the main driving factors in the process of N application affecting fungal community of wheat rhizosphere soil, with SOC being the most important factor [62]. In the present study, N application inhibited the microbial populations of rhizosphere soil by reducing the abundance of root exudates of *P. tabulaeformis*. This result was related to the negative response of root exudates to N application and the positive correlation between root exudates and microbial populations (Fig. 8). However, in the rhizosphere soil of *R. xanthina*, N application promoted root exudate abundances and inhibited the microbial community. This outcome was due to the relatively high content of carbohydrates in root exudates, and some secondary metabolites of carbohydrates were negatively correlated with microbial populations (Fig. 7). In the rhizosphere soil of *C. lancifolia*, the increase of soil nutrient contents caused by N application reduced root exudate abundance, so the microbial community was not promoted. This result reflects the mechanism that alleviating plant nutrient limitations would decrease carbon input from root exudates, a survival strategy that has been confirmed in studies on *Sibiraea angustata* and *Cunninghamia* [11, 12]. *C. lancifolia* exhibits this survival strategy, which is different from *P. tabulaeformis* and *R. xanthina*.

*P. tabulaeformis* roots yielded the most functional metabolites, including fatty acids, carbohydrates, nucleotides, and alcohols, *C. lancifolia* produced the fewest functional metabolites, which belong to fatty acids and alcohols. Different functional metabolites produced by plants had diverse effects on rhizosphere soil microbes [6, 63]. Our results confirmed that canopy trees possess the most functional metabolites in their root exudates, which alter the largest number of microbial populations of rhizosphere soil, followed by understory shrubs and grasses in forest ecosystems. Functional metabolites of root exudates exhibit species-specific traits, but the affected microbial populations are essentially the same among rhizosphere soils of different plant species. The affected bacterial genera mainly belong to *Proteobacteria*, *Actinobacteria*, and *Acidobacteria*, while fungal genera mainly belong to *Ascomycota* and *Basidiomycota*. This result may be related to the fact that *P. tabulaeformis*, *R. xanthina*, and *C. lancifolia* grow in the same type of soil. In addition, our study found that root exudates only have promotive effects on fungal populations but exhibited both promotive and inhibitory effects on bacterial populations. Wang et al. [64] revealed that there were generally positive relationships between fungal populations and soil properties, but multiple relationships between bacterial populations and soil properties in subalpine forests. Soil fungal populations exhibit a mostly consistent response to environmental changes due to the low diversity, whereas bacterial populations have varied responses to environmental changes due to the high diversity. This may be the reason that relationships between bacterial and fungal populations and functional metabolites in this study were quite different. By using untargeted metabolomics methods, we determined that 2-Hydroxypyridine (alcohol), Palmitic acid (fatty acid), and Stearic acid (stearic acid) were the main functional metabolites affecting rhizosphere soil microbes in *P. tabulaeformis* forests, thus these metabolites will be the focus of our subsequent research.

## Conclusion

Canopy trees had higher relative abundance of root exudates and a quiet different composition compared to understory shrubs and grasses in forest ecosystem. After N application, the relative abundances of root exudates of *P. tabulaeformis* and *R. xanthina* exhibited parabolic changes, while that of *C. lancifolia* showed a decreasing trend. Microbial quantity in the rhizosphere soil of *C. lancifolia* was higher than those of *P. tabulaeformis* and *R. xanthina*, while microbial diversity remained relatively unchanged. Soil bacterial and fungal quantities were enhanced and inhibited by N application, respectively, while microbial diversity was promoted. Through the PLS-PM model, we identified different mechanisms by which trees, shrubs, and grasses affect the microbial community of rhizosphere soil in response to N application. Root exudates and soil chemical properties played different roles in these mechanisms, indicating varied survival strategies of plant species. The carbon-free nutrient solution used for exudate collection would augment additional stress for plant root, which is inconsistent with the natural environment. Therefore, further studies should design the carbon-free nutrient solution based on the soil chemical properties, and reduce operational disturbances by taking multiple measurements as long as methods and materials allow.

### Supplementary information

Figure S1

Figure S2

Figure S3

Supplementary methods

## Data Availability

High-throughput sequencing data are provided at the NCBI (SRA) database under the study accession code PRJNA1007781. All other datasets are available upon request.

## References

[1] Yuan Y, Dai X, Fu X, Kou L, Luo Y, Jiang L (2020). Differences in the rhizosphere effects among trees, shrubs and herbs in three subtropical plantations and their seasonal variations. Eur J Soil Biol.

[2] Nakayama M, Tateno R (2022). Rhizosphere effects on soil extracellular enzymatic activity and microbial abundance during the low-temperature dormant season in a northern hardwood forest. Rhizosphere.

[3] Ambus P, Robertson GP (2006). The effect of increased n deposition on nitrous oxide, methane and carbon dioxide fluxes from unmanaged forest and grassland communities in Michigan. Biogeochemistry.

[4] Raich JW, Tufekciogul AJB (2000). Vegetation and soil respiration: Correlations and controls. Biogechemistry.

[5] Mayerhofer MM, Eigemann F, Lackner C, Hoffmann J, Hellweger FL (2021). Dynamic carbon flux network of a diverse marine microbial community. ISME Commun.

[6] Yoneyama K, Xie XN, Kim HI, Kisugi T, Nomura T, Sekimoto H (2012). How do nitrogen and phosphorus deficiencies affect strigolactone production and exudation?. Planta.

[7] Kuang J, Han S, Chen Y, Bates CT, Wang P, Shu W (2021). Root-associated fungal community reflects host spatial co-occurrence patterns in a subtropical forest. ISME Commun.

[8] Wu R, Cheng X, Zhou W, Han H (2019). Microbial regulation of soil carbon properties under nitrogen addition and plant inputs removal. Peerj.

[9] Yang J, Wang X, Sun L, Wang C, Bai E (2020). Effects of nitrogen and phosphorus addition on soil microbial community and amino sugar in a temperate forest on Changbai Mountain, Northeast China. J Appl Ecol.

[10] Bloom AJ, Chapin FS, Mooney HA (1985). Resource limitation in plants-an economic analogy. Annu Rev Ecol Syst.

[11] Xiong D, Huang J, Yang Z, Cai Y, Lin T, Liu X (2020). The effects of warming and nitrogen addition on fine root exudation rates in a young Chinese-fir stand. For Ecol Manage.

[12] He W, Yang X, Xiao J, Zhang Z, Jiang Z, Yuan Y (2017). Effects of nitrogen enrichment on root exudative carbon inputs in *Sibiraea angustata* shrubbery at the eastern fringe of Qinghai-Xizang Plateau. Chin J Plant Ecol.

[13] Xu G, Li S, Zhao Y, Chen M, Li Y (2014). Effects of straw returning and nitrogen fertilizer application on root secretion and nitrogen utilization of rice. Acta Prataculturae Sinica.

[14] Burrill HM, Wang G, Bever JD (2023). Rapid differentiation of soil and root microbiomes in response to plant composition and biodiversity in the field. ISME Commun.

[15] Zhu S, Vivanco JM, Manter DK (2016). Nitrogen fertilizer rate affects root exudation, the rhizosphere microbiome and nitrogen-use-efficiency of maize. Appl Soil Ecol.

[16] Weng B, Xie X, Yang J, Liu J, Lu H, Yan C (2013). Research on the nitrogen cycle in rhizosphere of *Kandelia obovata* under ammonium and nitrate addition. Mar Pollut Bull.

[17] Zhai D, Jin W, Shao J, He Y, Zhang G, Li M (2017). Different response patterns of soil respiration to a nitrogen addition gradient in four types of land-use on an alluvial island in China. Ecosystems.

[18] Ma S, Chen G, Tian D, Du E, Xiao W, Jiang L (2020). Effects of seven-year nitrogen and phosphorus additions on soil microbial community structures and residues in a tropical forest in Hainan Island, China. Geoderma.

[19] Zhao X, Li Y, Xie Z, Li P (2020). Effects of nitrogen deposition and plant litter alteration on soil respiration in a semiarid grassland. Sci Total Environ.

[20] Phillips RP, Erlitz Y, Bier R, Bernhardt ES (2008). New approach for capturing soluble root exudates in forest soils. Funct Ecol.

[21] Proctor C, He Y (2017). Quantifying root extracts and exudates of sedge and shrub in relation to root morphology. Soil Biol Biochem.

[22] Sun L, Ataka M, Han M, Han Y, Gan D, Xu T (2020). Root exudation as a major competitive fine-root functional trait of 18 coexisting species in a subtropical forest. New Phytol.

[23] Caporaso JG, Kuczynski J, Stombaugh J, Bittinger K, Bushman FD, Costello EK (2010). QIIME allows analysis of high-throughput community sequencing data. Nat Methods.

[24] Lundberg DS, Lebeis SL, Paredes SH, Yourstone S, Gehring J, Malfatti S (2012). Defining the core Arabidopsis thaliana root microbiome. Nature.

[25] DeSantis TZ, Hugenholtz P, Larsen N, Rojas M, Brodie EL, Keller K (2006). Greengenes, a chimera-checked 16S rRNA gene database and workbench compatible with ARB. Appl Environ Microbiol.

[26] Katoh K, Misawa K, Kuma K, Miyata T (2002). MAFFT: a novel method for rapid multiple sequence alignment based on fast Fourier transform. Nucleic Acids Res.

[27] Price MN, Dehal PS, Arkin AP (2009). FastTree: computing large minimum evolution trees with profiles instead of a distance matrix. Mol Biol Evol.

[28] Mebius LJ (1960). A rapid method for the determination of organic carbon in soil. Anal Chim Acta.

[29] Page AL, Miller RH, Dennis RK (1982). Methods of soil analysis. Am Soc Agronomy: Soil Sci Soc Am.

[30] Lu, R. Methods for soil agrochemistry analysis. Agric Sci Technol Press, Beijing China, 2000. pp 106–310.

[31] Lu S, Hu H, Sun Y, Yang J (2009). Study on the growth characteristics and root exudates of three wetlands plants at different culture conditions. Chin J Environ Sci.

[32] Jiang C, Shimono M, Maeda S, Inoue H, Mori M, Hasegawa M (2009). Suppression of the rice fatty-acid desaturase gene OsSSI2 enhances resistance to blast and leaf blight diseases in rice. Mol Plant-Microbe Interact.

[33] Kachroo A, Kachroo P (2009). Fatty acid-derived signals in plant defense. Annu Rev Phytopathol.

[34] Carreras A, Bernard S, Durambur G, Gugi B, Loutelier C, Pawlak B (2020). In vitro characterization of root extracellular trap and exudates of three Sahelian woody plant species. Planta.

[35] Li W, Wang J, Li X, Wang S, Liu W, Shi S (2019). Nitrogen fertilizer regulates soil respiration by altering the organic carbon storage in root and topsoil in alpine meadow of the north-eastern Qinghai-Tibet Plateau. Sci Rep.

[36] Strehmel N, Bottcher C, Schmidt S, Scheel D (2014). Profiling of secondary metabolites in root exudates of *Arabidopsis thaliana*. Phytochemistry.

[37] Wang Y, Ren W, Li Y, Xu Y, Teng Y, Christie P (2019). Nontargeted metabolomic analysis to unravel the impact of di (2-ethylhexyl) phthalate stress on root exudates of alfalfa (*Medicago sativa*). Sci Total Environ..

[38] Berg G, Smalla K (2009). Plant species and soil type cooperatively shape the structure and function of microbial communities in the rhizosphere. FEMS Microbiol Ecol.

[39] Dakora FD, Phillips DA (2002). Root exudates as mediators of mineral acquisition in low-nutrient environments. Plant Soil.

[40] Jones D, Darrah P (1994). Amino-acid influx at the soil-root interface of *Zea mays* L. and its implications in the rhizosphere. Plant Soil.

[41] Shepherd T, Davies HV (1994). Patterns of short-term amino acid accumulation and loss in the root-zone of liquid-cultured forage rape (*Brassica napus* L.). Plant Soil.

[42] Hussain Q, Pan G, Liu Y, Zhang A, Li L, Zhang X (2012). Microbial community dynamics and function associated with rhizosphere over periods of rice growth. Plant Soil Environ.

[43] Dai Y, Hou X, Yan Z, Wu H, Xie J, Zhang X (2016). Soil microbes and the chemical properties of the rhizosphere and non-rhizosphere soil under two types of vegetation restoration in the hobq sandy land of inner mongolia,China. Acta Ecol Sin.

[44] Hamer U, Makeschin F (2009). Rhizosphere soil microbial community structure and microbial activity in set-aside and intensively managed arable land. Plant Soil.

[45] Dennis PG, Miller AJ, Hirsch PR (2010). Are root exudates more important than other sources of rhizodeposits in structuring rhizosphere bacterial communities?. FEMS Microbiol Ecol.

[46] Benizri E, Dedourge O, Dibattista-Leboef C, Piutti S, Nguyen C, Guckert A (2002). Effect of maize rhizodeposits on soil microbial community structure. Appl Soil Ecol.

[47] Liu Y, Zhang H, Xiong M, Li F, Li L, Wang G (2017). Abundance and composition response of wheat field soil bacterial and fungal communities to elevated CO_2_ and increased air temperature. Biol Fertil Soils.

[48] Su Y, Huang G, Lin Y, Zhang Y (2016). No synergistic effects of water and nitrogen addition on soil microbial communities and soil respiration in a temperate desert. Catena.

[49] Singh BK, Munro S, Potts JM, Millard P (2007). Influence of grass species and soil type on rhizosphere microbial community structure in grassland soils. Appl Soil Ecol.

[50] Kuang X, Si K, Song H, Peng L, Chen A (2021). Lime-phosphorus fertilizer efficiently reduces the Cd content of rice: physicochemical property and biological community structure in Cd-polluted paddy soil. Front Microbiol.

[51] Srinivasan S, Lee JJ, Kim MK (2011). *Sphingomonas rosea* sp. nov. and *Sphingomonas swuensis* sp. nov., rosy colored *β*-glucosidase-producing bacteria isolated from soil. J Microbiol.

[52] Chen L, He Z, Wu X, Du J, Zhu X, Lin P (2021). Linkages between soil respiration and microbial communities following afforestation of alpine grasslands in the northeastern Tibetan Plateau. Appl Soil Ecol.

[53] Hou F, Du J, Bi X, Yuan Y, Wu X (2022). Toxicity effects of aged refuse on *Tagetes patula* and rhizosphere microbes. Land Degrad Dev.

[54] Li W, Zhang Y, Mao W, Wang C, Yin S (2020). Functional potential differences between *Firmicutes* and *Proteobacteria* in response to manure amendment in a reclaimed soil. Can J Microbiol.

[55] Liu L, Greaver T (2010). A global perspective on belowground carbon dynamics under nitrogen enrichment. Ecol Lett.

[56] Han Y, Feng J, Han M, Zhu B (2020). Responses of arbuscular mycorrhizal fungi to nitrogen addition: A meta-analysis. Global Change Biol.

[57] Preusser S, Poll C, Marhan S, Angst G, Mueller CW, Bachmann J (2019). Fungi and bacteria respond differently to changing environmental conditions within a soil profile. Soil Biol Biochem.

[58] Wang C, Smith GR, Gao C, Peay KG (2023). Dispersal changes soil bacterial interactions with fungal wood decomposition. ISME Commun.

[59] Luo P, Han X, Wang Y, Han M, Shi H, Liu N (2015). Influence of long-term fertilization on soil microbial biomass, dehydrogenase activity, and bacterial and fungal community structure in a brown soil of northeast China. Ann Microbiol.

[60] Raczka NC, Carrara JE, Brzostek ER (2022). Plant-microbial responses to reduced precipitation depend on tree species in a temperate forest. Global Change Biol.

[61] Wang J, Liao L, Wang G, Liu H, Wu Y, Liu G (2022). N-induced root exudates mediate the rhizosphere fungal assembly and affect species coexistence. Sci Total Environ.

[62] Chen S, Waghmode TR, Sun R, Kuramae EE, Hu C, Liu B (2019). Root-associated microbiomes of wheat under the combined effect of plant development and nitrogen fertilization. Microbiome.

[63] Xing J, Chin CK (2000). Modification of fatty acids in eggplant affects its resistance to *Verticillium dahliae*. Physiol Mol Plant P.

[64] Wang Z, Bai Y, Hou J, Li F, Li X, Cao R (2022). The changes in soil microbial communities across a subalpine forest successional series. Forests.

