# Supplementary material for: The mechanism effects of root exudate on microbial community of rhizosphere soil of tree, shrub, and grass in forest ecosystem under N deposition
Source: ISME Commun. 2023 Nov 20;3:120. doi: 10.1038/s43705-023-00322-9 (PMC10662252; doi:10.1038/s43705-023-00322-9)
Supplement: Supplementary file 1 — Figure S1 [file 43705_2023_322_MOESM1_ESM.pdf]

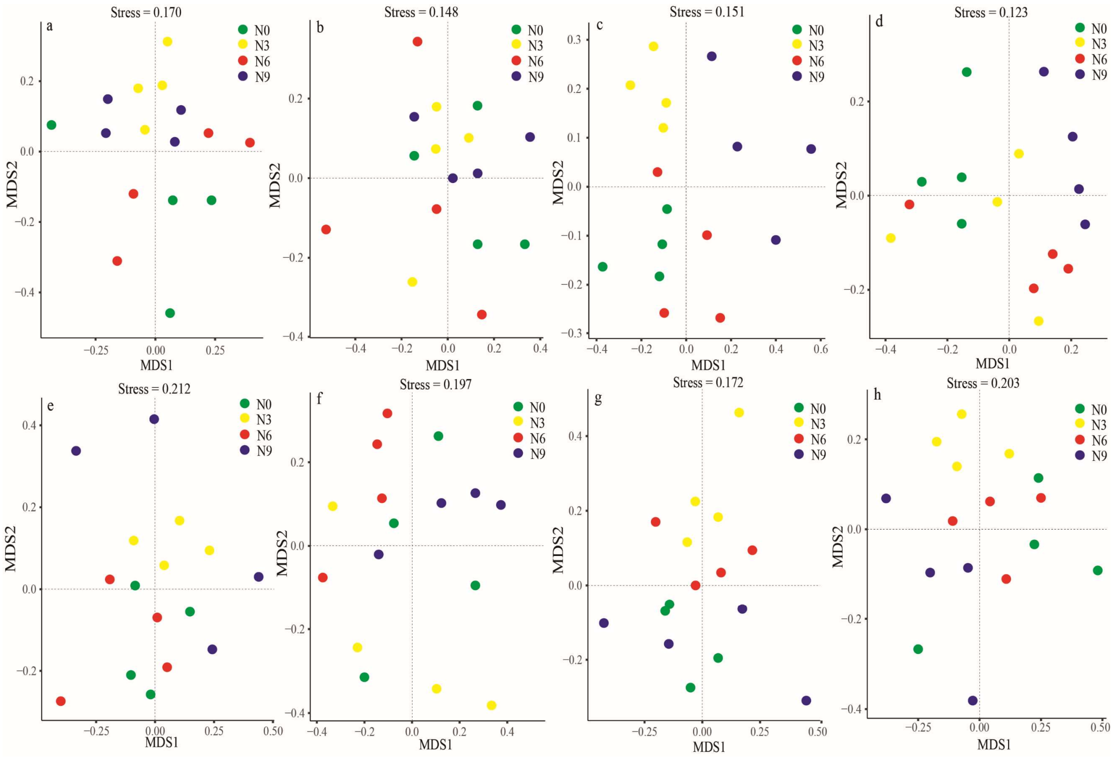

Fig. S1 NMDS analysis plot based on Bray-Curtis distances depicting the compositions of bacterial community in rhizosphere soil of *P. tabulaeformis* (a), *R. xanthina* (b), *C. lancifolia* (c), non-rhizosphere soil (d), and fungal community in the rhizosphere soil of *P. tabulaeformis* (e), *R. xanthina* (f), *C. lancifolia* (g), and non-rhizosphere soil (f) across the N application treatments.

Note: N0, N3, N6, N9 are 0, 3, 6, and 9 g N m^{-2} y^{-1} respectively.
