# Supplementary material for: The mechanism effects of root exudate on microbial community of rhizosphere soil of tree, shrub, and grass in forest ecosystem under N deposition
Source: ISME Commun. 2023 Nov 20;3:120. doi: 10.1038/s43705-023-00322-9 (PMC10662252; doi:10.1038/s43705-023-00322-9)
Supplement: Supplementary file 2 — Figure S2 [file 43705_2023_322_MOESM2_ESM.pdf]

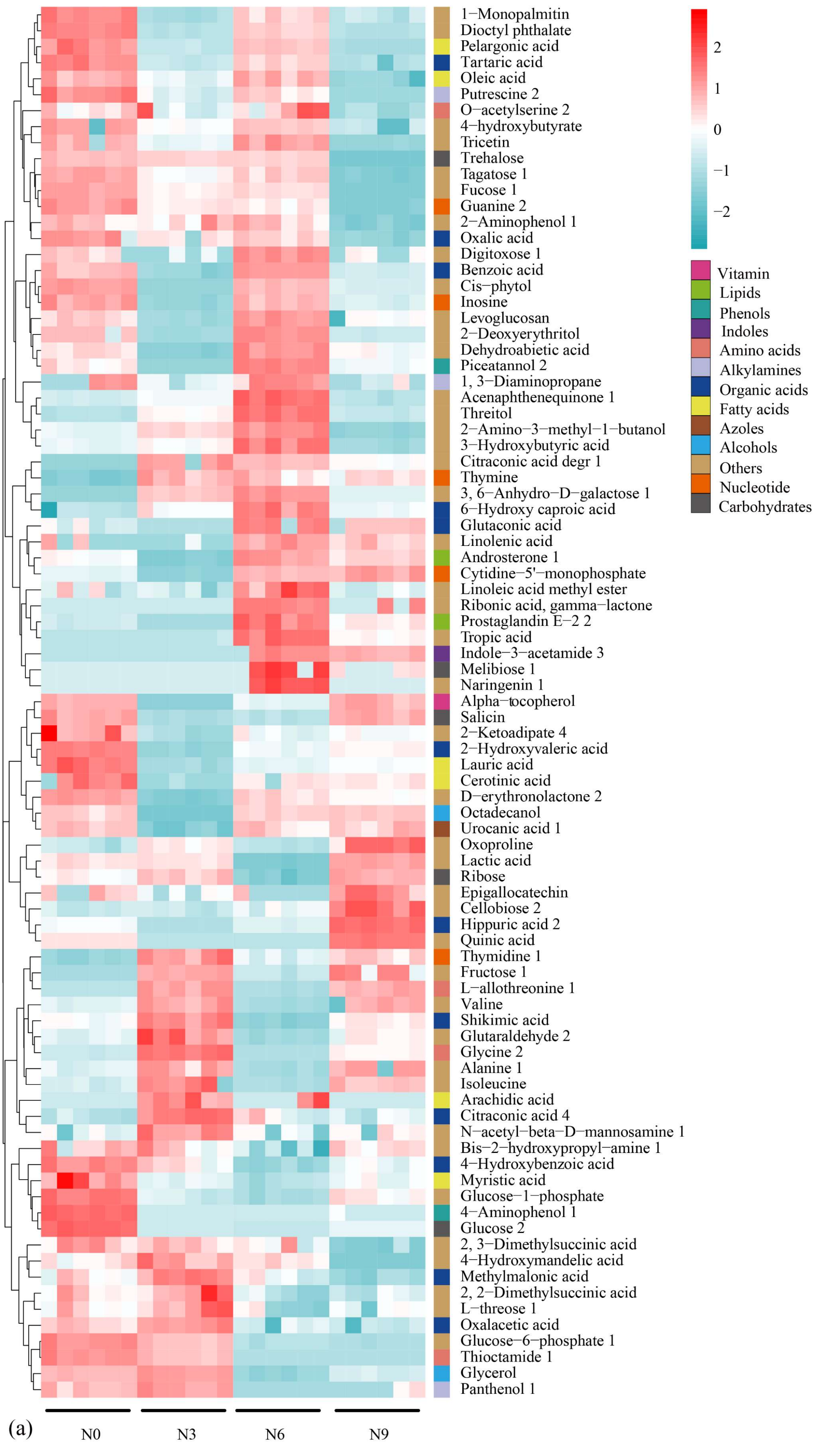
1−Monopalmitin
Dioctyl phthalate
Pelargonic acid
Tartaric acid
Oleic acid
Putrescine 2
O−acetylserine 2
4−hydroxybutyrate
Tricetin
Trehalose
Tagatose 1
Fucose 1
Guanine 2
2−Aminophenol 1
Oxalic acid
Digitoxose 1
Benzoic acid
Cis−phytol
Inosine
Levoglucosan
2−Deoxyerythritol
Dehydroabietic acid
Piceatannol 2
1, 3−Diaminopropane
Acenaphthenequinone 1
Threitol
2−Amino−3−methyl−1−butanol
3−Hydroxybutyric acid
Citraconic acid degr 1
Thymine
3, 6−Anhydro−D−galactose 1
6−Hydroxy caproic acid
Glutaconic acid
Linolenic acid
Androsterone 1
Cytidine−5'−monophosphate
Linoleic acid methyl ester
Ribonic acid, gamma−lactone
Prostaglandin E−2 2
Tropic acid
Indole−3−acetamide 3
Melibiose 1
Naringenin 1
Alpha−tocopherol
Salicin
2−Ketoadipate 4
2−Hydroxyvaleric acid
Lauric acid
Cerotinic acid
D−erythronolactone 2
Octadecanol
Urocanic acid 1
Oxoproline
Lactic acid
Ribose
Epigallocatechin
Cellobiose 2
Hippuric acid 2
Quinic acid
Thymidine 1
Fructose 1
L−allothreonine 1
Valine
Shikimic acid
Glutaraldehyde 2
Glycine 2
Alanine 1
Isoleucine
Arachidic acid
Citraconic acid 4
N−acetyl−beta−D−mannosamine 1
Bis−2−hydroxypropyl−amine 1
4−Hydroxybenzoic acid
Myristic acid
Glucose−1−phosphate
4−Aminophenol 1
Glucose 2
2, 3−Dimethylsuccinic acid
4−Hydroxymandelic acid
Methylmalonic acid
2, 2−Dimethylsuccinic acid
L−threose 1
Oxalacetic acid
Glucose−6−phosphate 1
Thioctamide 1
Glycerol
Panthenol 1
2
1
0
−1
−2
Vitamin
Lipids
Phenols
Indoles
Amino acids
Alkylamines
Organic acids
Fatty acids
Azoles
Alcohols
Others
Nucleotide
Carbohydrates
N0
N3
N6
N9
(a)

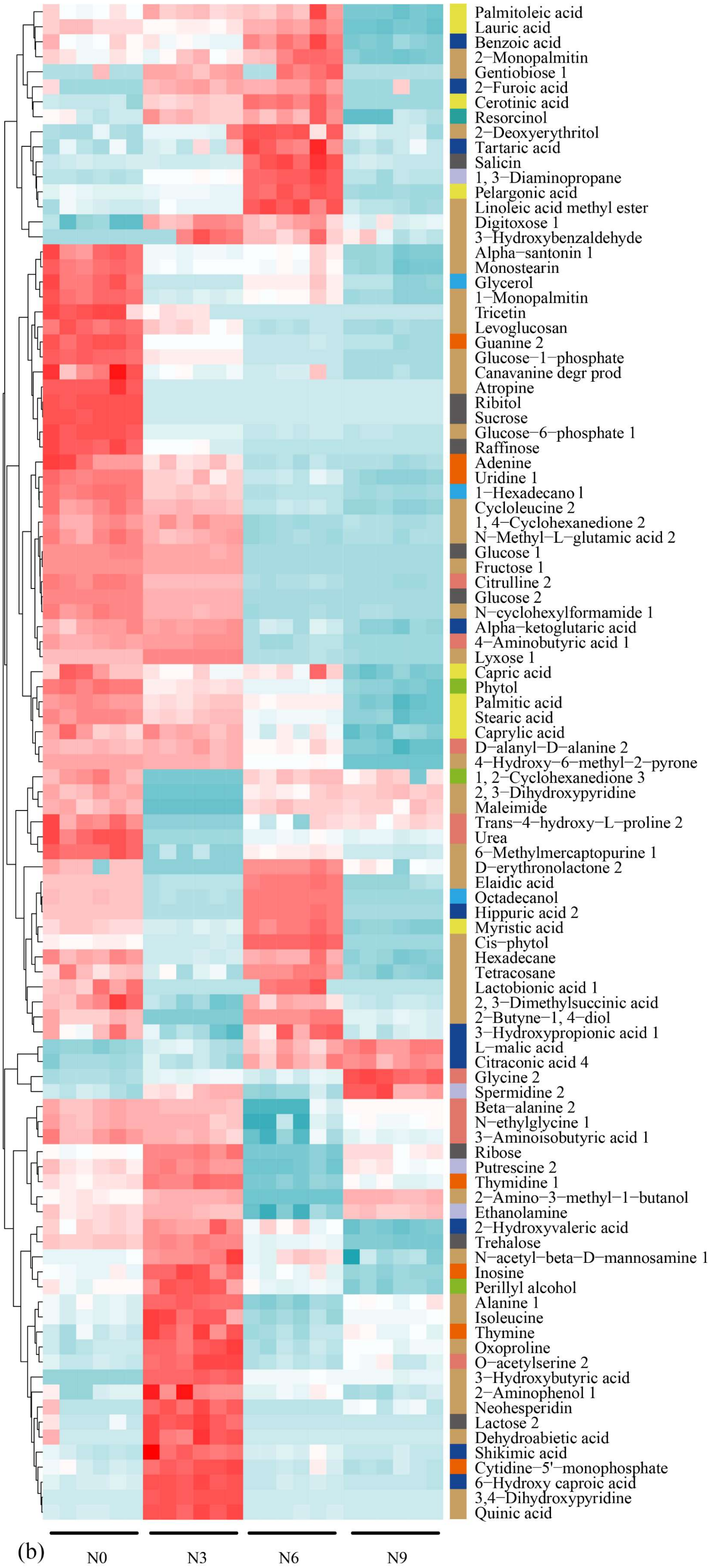

Palmitoleic acid
Lauric acid
Benzoic acid
2−Monopalmitin
Gentiobiose 1
2−Furoic acid
Cerotinic acid
Resorcinol
2−Deoxyerythritol
Tartaric acid
Salicin
1, 3−Diaminopropane
Pelargonic acid
Linoleic acid methyl ester
Digitoxose 1
3−Hydroxybenzaldehyde
Alpha−santonin 1
Monostearin
Glycerol
1−Monopalmitin
Tricetin
Levoglucosan
Guanine 2
Glucose−1−phosphate
Canavanine degr prod
Atropine
Ribitol
Sucrose
Glucose−6−phosphate 1
Raffinose
Adenine
Uridine 1
1−Hexadecano l
Cycloleucine 2
1, 4−Cyclohexanedione 2
N−Methyl−L−glutamic acid 2
Glucose 1
Fructose 1
Citrulline 2
Glucose 2
N−cyclohexylformamide 1
Alpha−ketoglutaric acid
4−Aminobutyric acid 1
Lyxose 1
Capric acid
Phytol
Palmitic acid
Stearic acid
Caprylic acid
D−alanyl−D−alanine 2
4−Hydroxy−6−methyl−2−pyrone
1, 2−Cyclohexanedione 3
2, 3−Dihydroxypyridine
Maleimide
Trans−4−hydroxy−L−proline 2
Urea
6−Methylmercaptopurine 1
D−erythronolactone 2
Elaidic acid
Octadecanol
Hippuric acid 2
Myristic acid
Cis−phytol
Hexadecane
Tetracosane
Lactobionic acid 1
2, 3−Dimethylsuccinic acid
2−Butyne−1, 4−diol
3−Hydroxypropionic acid 1
L−malic acid
Citraconic acid 4
Glycine 2
Spermidine 2
Beta−alanine 2
N−ethylglycine 1
3−Aminoisobutyric acid 1
Ribose
Putrescine 2
Thymidine 1
2−Amino−3−methyl−1−butanol
Ethanolamine
2−Hydroxyvaleric acid
Trehalose
N−acetyl−beta−D−mannosamine 1
Inosine
Perillyl alcohol
Alanine 1
Isoleucine
Thymine
Oxoproline
O−acetylserine 2
3−Hydroxybutyric acid
2−Aminophenol 1
Neohesperidin
Lactose 2
Dehydroabietic acid
Shikimic acid
Cytidine−5'−monophosphate
6−Hydroxy caproic acid
3,4−Dihydroxypyridine
Quinic acid
N0
N3
N6
N9

(b)

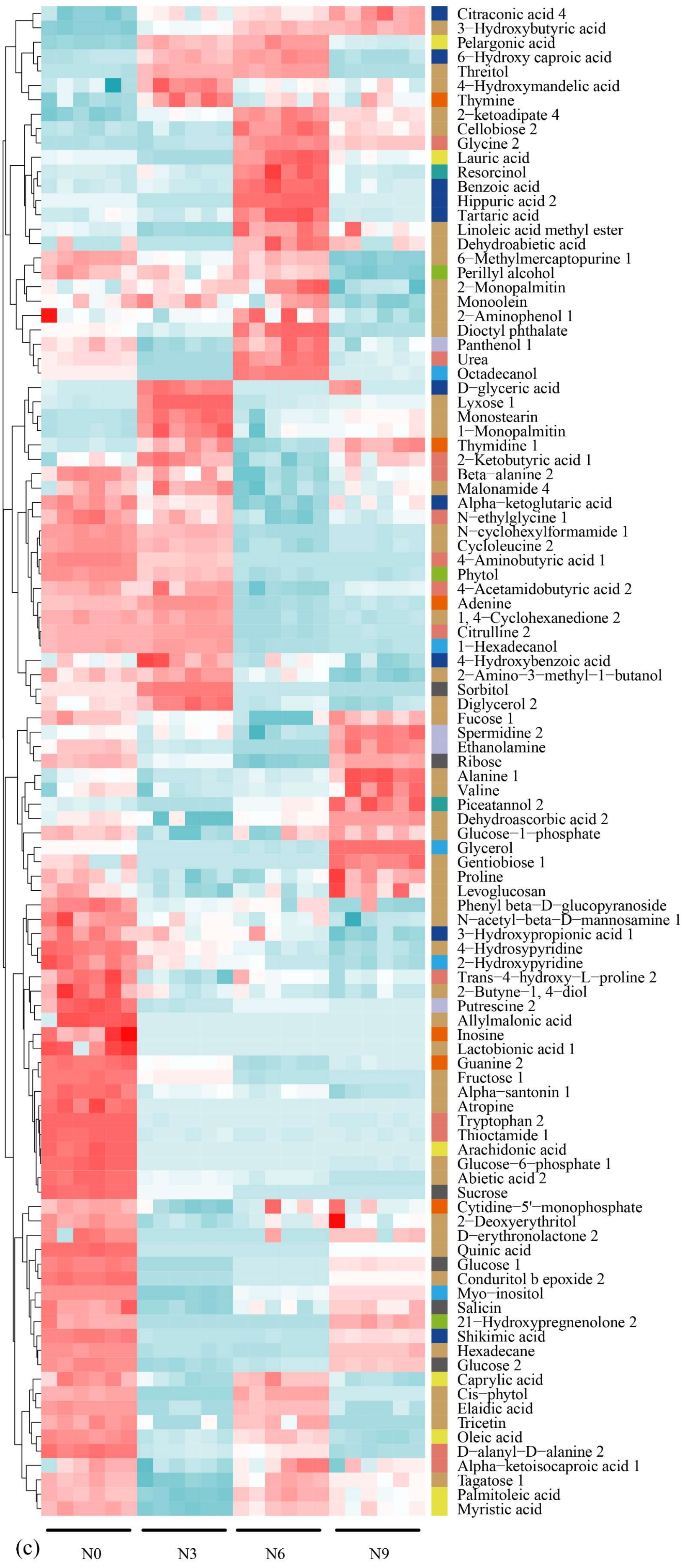
Citraconic acid 4
3−Hydroxybutyric acid
Pelargonic acid
6−Hydroxy caproic acid
Threitol
4−Hydroxymandelic acid
Thymine
2−ketoadipate 4
Cellobiose 2
Glycine 2
Lauric acid
Resorcinol
Benzoic acid
Hippuric acid 2
Tartaric acid
Linoleic acid methyl ester
Dehydroabietic acid
6−Methylmercaptopurine 1
Perillyl alcohol
2−Monopalmitin
Monoolein
2−Aminophenol 1
Dioctyl phthalate
Panthenol 1
Urea
Octadecanol
D−glyceric acid
Lyxose 1
Monostearin
1−Monopalmitin
Thymidine 1
2−Ketobutyric acid 1
Beta−alanine 2
Malonamide 4
Alpha−ketoglutaric acid
N−ethylglycine 1
N−cyclohexylformamide 1
Cycloleucine 2
4−Aminobutyric acid 1
Phytol
4−Acetamidobutyric acid 2
Adenine
1, 4−Cyclohexanedione 2
Citrulline 2
1−Hexadecanol
4−Hydroxybenzoic acid
2−Amino−3−methyl−1−butanol
Sorbitol
Diglycerol 2
Fucose 1
Spermidine 2
Ethanolamine
Ribose
Alanine 1
Valine
Piceatannol 2
Dehydroascorbic acid 2
Glucose−1−phosphate
Glycerol
Gentiobiose 1
Proline
Levoglucosan
Phenyl beta−D−glucopyranoside
N−acetyl−beta−D−mannosamine 1
3−Hydroxypropionic acid 1
4−Hydrosypyridine
2−Hydroxypyridine
Trans−4−hydroxy−L−proline 2
2−Butyne−1, 4−diol
Putrescine 2
Allylmalonic acid
Inosine
Lactobionic acid 1
Guanine 2
Fructose 1
Alpha−santonin 1
Atropine
Tryptophan 2
Thioctamide 1
Arachidonic acid
Glucose−6−phosphate 1
Abietic acid 2
Sucrose
Cytidine−5'−monophosphate
2−Deoxyerythritol
D−erythronolactone 2
Quinic acid
Glucose 1
Conduritol b epoxide 2
Myo−inositol
Salicin
21−Hydroxypregnenolone 2
Shikimic acid
Hexadecane
Glucose 2
Caprylic acid
Cis−phytol
Elaidic acid
Tricetin
Oleic acid
D−alanyl−D−alanine 2
Alpha−ketoisocaproic acid 1
Tagatose 1
Palmitoleic acid
Myristic acid
(c)
N0
N3
N6
N9

Fig. S2 Clustered heatmap of the differential root exudates and their associated primary metabolites across the N application treatments at different plant species (VIP > 2.0 and $p < 0.05$).

Note: a, root exudate profiles across the N application treatments in *P. tabulaeformis* root; b, root exudate profiles across the N application treatments in *R. xanthina* root; c, root exudate profiles across the N application treatments in *C. lancifolia* root. N0, N3, N6, N9 are 0, 3, 6, and 9 g N m^{-2} y^{-1} respectively.
