# Supplementary material for: The mechanism effects of root exudate on microbial community of rhizosphere soil of tree, shrub, and grass in forest ecosystem under N deposition
Source: ISME Commun. 2023 Nov 20;3:120. doi: 10.1038/s43705-023-00322-9 (PMC10662252; doi:10.1038/s43705-023-00322-9)
Supplement: Supplementary file 3 — Figure S3 [file 43705_2023_322_MOESM3_ESM.pdf]

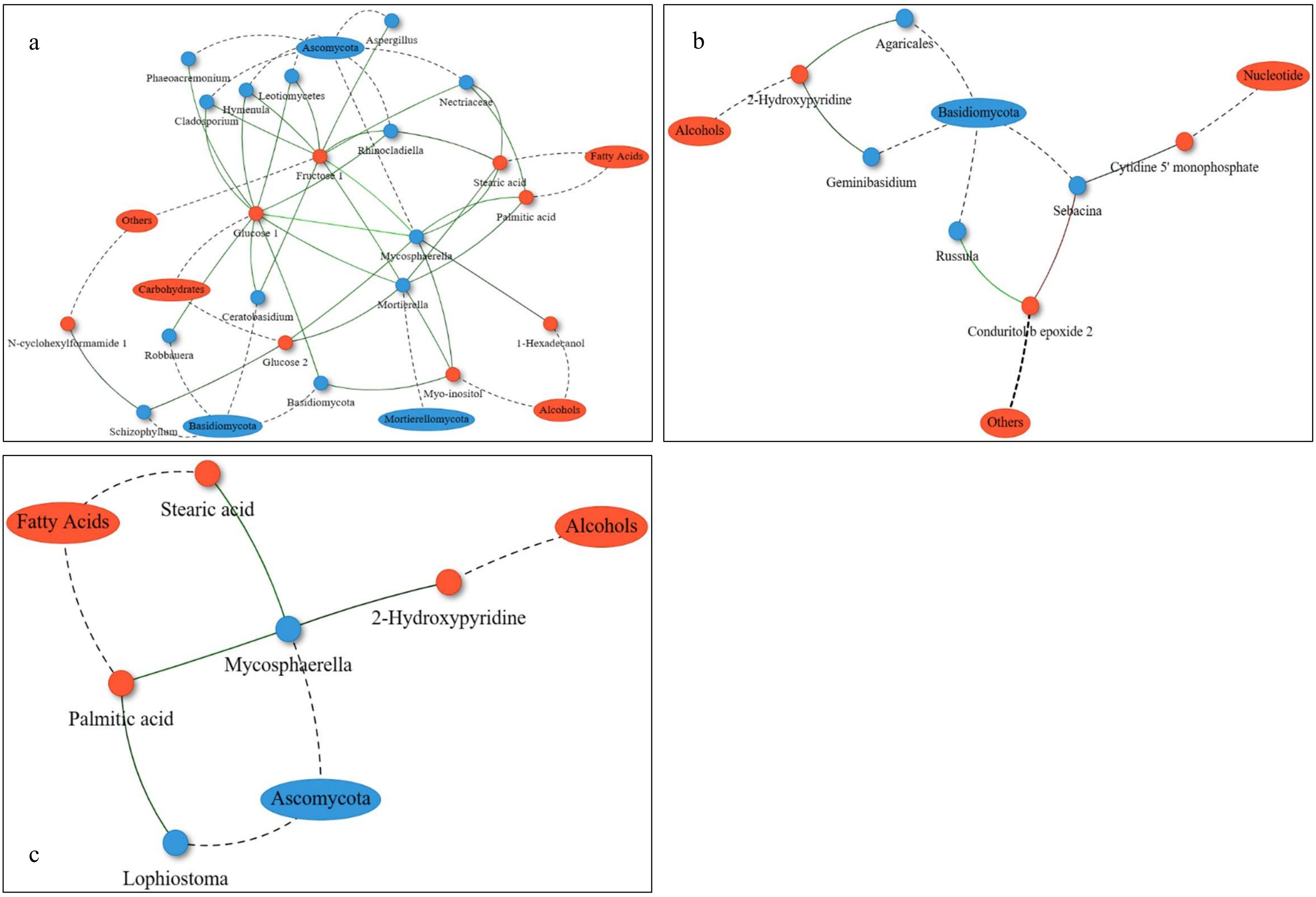

Fig. S3 Network of correlation analysis between differential exudates and fungal populations across N application treatments.

Note: Only significant correlations are shown ($p < 0.05$). The green lines show positive correlations, the red lines show negative correlations, and the dashed lines show the relationship between secondary and primary metabolites, and the relationship between genus and phylum. a, correlation analysis between root exudates and fungal populations of *P. tabulaeformis*; b, correlation analysis between root exudates and fungal populations of *R. xanthina*; c, correlation analysis between exudates and fungal populations of *C. lancifolia*.
