# Supplementary material for: The mechanism effects of root exudate on microbial community of rhizosphere soil of tree, shrub, and grass in forest ecosystem under N deposition
Source: ISME Commun. 2023 Nov 20;3:120. doi: 10.1038/s43705-023-00322-9 (PMC10662252; doi:10.1038/s43705-023-00322-9)
Supplement: Supplementary file 4 — Supplementary methods [file 43705_2023_322_MOESM4_ESM.docx]

**SUPPLEMENTARY METHODS**

**Amplicon sequencing analysis**

PCR was used to evaluate the microbial quantity of bacteria and fungi. Total genomic DNA samples were extracted from 0.25g homogenized soil using the power soil separation kit (MOBIO Laboratories, Inc. Carlsbad, CA), following the manufacturer’s instructions, and stored at -20°C prior to further analysis. Forward primer 338f (50ACTCCTACGGGAGGCAGCAG) and reverse primer 518r (50ATTACCGCGGCTGCTGG) were used for PCR amplification of the bacterial 16S rRNA genes V3-V4 region [1]. Meanwhile, 18s ITS 1f (50 CTTGGTCATTTAGAGGAAGTAA) and 18s ITS 4r (50 CATGGAGACTTGTACACGGTCCAG) were used for the identification of fungi [2]. The brief operations are described as follows. Veriti 96-well thermal cycler (Applied Biosystems) was used for PCR gradient set, and then high temperature annealing were operated, the formal PCR was performed on an ABI 7500 Real Time PCR system (Applied Biosystems, Foster City, CA). PCR amplification includes denaturation at 92°C for 2 min, protein denaturation 30 times at 92°C for 1 min, annealing at 55°C for 30 s, amplification at 72°C for 1 min, and final amplification at 72°C for 6 min. The 20 ml extract solution was composed by 10 ml premix, 10 pmol primers, 6 ml sterilized nuclease-free water and 2 ml template DNA. After the PCR amplification, copy numbers of each PCR was calculated based on the standard curves. Standard curves were obtained by preparing standards with PCR products extracted and purified from homogenized soil, and R^2^ was required to be greater than 0.995. The inhibition of amplification was evaluated according to Dumonceaux et al. [3], and the specificity was checked by melting curve analysis.

Sequencing was performed on the Illumina MiSeqPE300 platform. Firstly, the target fragment was amplified by PCR. The V3-V4 region of bacterial 16S rRNA gene was performed with forward primer 341F (CCTACGGGNGGCWGCAG) and reverse primer 805R (GACTACHVGGGTATCTAATCC). The primers used for PCR amplification of fungal internal transcription were GC-clamp ITS1F and ITS2 [4, 5]. Bacterial amplification was performed using rTaq DNA polymerase (Takara, Shiga, Japan) in a 20 μL volume containing buffer (2 μL), dNTPs (2.5 mM of 2 μL), forward primer (5 μM of 0.8 μL), reverse primer (5 μM of 0.8 μL), rTaq polymerase (0.2 μL), bovine serum albumin (BSA, 0.2 μL), and template DNA (10 ng), sterilized nuclease-free water was used to replenish the remaining volume. PCR analysis was performed in GeneAmp PCR System 9700 (ABI 9700, PerkinElmer, USA) with the following operations: denaturation at 95°C for 3 min; protein denaturation 27 times at 95°C for 30 s; annealed at 55°C for 30 s; amplified at 72°C for 45 s and final amplification at 72°C for 10 min. During the amplification of fungal PCR, the mixture was composed by template DNA (10 ng), MgCl_2_ (1 mM), GoTap Green master mixture (Promega, Fitchburg, WI, USA), and primers (5 pmol of each). The reaction conditions include denaturation at 95°C for 2 min; protein denaturation 35 times at 95°C for 30 s; annealed at 55°C for 30 s; amplified at 72°C for 60 s and final amplification at 72°C for 6 min. After that, the quality and concentration of PCR amplification were verified using 2% agarose gel electrophoresis and QuantiFluor™-ST fluorometer (Promega, Fitchburg, WI, USA) respectively, and sequencing was performed on an Illumina MiSeqPE300 (Illumina, San Diego, CA, USA) platform. The number and length of bacteria were 30,000 and 441 bp respectively, while those of fungi were 32,000 and 270 bp respectively.

**Non-targeted metabolomics analysis of root exudates**

The composition and relative abundance of root exudate of three plant species were tested by non-targeted metabolomics analysis, based on the gas chromatography-time-of-flight mass spectrometry, and the main operations included exudate extraction, machine detection and off-line data processing. 30 mL root exudate sample was freeze-dried in a 50 mL EP tube, 1 mL methanol was added for pre-cold extraction, and ultrasonic treatment in ice water bath for 15 min. After that, sample was centrifuged at 10000 rpm for 15 min, and 1.5 mL supernatant was transferred to a 2 mL EP tube. Meanwhile, the reliability of the determination operations were checked using quality control samples (QC), which were composed by mixing 400 μL of each supernatant. Supernatant samples and QC were dried in a vacuum concentrator, and incubated at 80°C for 30 min with methoxamine salt solution (methoxamine hydrochloride in 20 mg Ml^-1^ pyridine). Then, BSTFA solution (containing 1% TMCS, v/v) was mixed with supernatant sample and incubated at 70 °C for 1.5 h, and 5 μL FAMEs (dissolved in chloroform) was added for machine detection after cooling. Machine detection was performed on the gas chromatography-time-of-flight mass spectrometry (Agilent 7890，USA), and the capillary column model was Agilent DB-5MS (30 m × 250 μm × 0.25 μm, J&W Scientific, Folsom, CA, USA). Parameter of machine detection included the single injection volume was 1 μL, the flow rate of inlet pad was 3 ml min^-1^, the flow rate of column was 1 mL min^-1^, the temperature of column chamber was kept at 50 °C for 1 min, and then the temperature was increased to 310 °C at 10°C min^-1^ for 8 min, the sample inlet temperature was 280 °C, the transfer tube temperature was 280 °C, the ion source temperature was 250 °C, and the solvent delay time was 6.35 min. The state of the machine and data quality were monitored during sample testing. The relative standard deviation of QC samples was 8.34%, which indicated that the machine detection system had good stability. During off-line data processing, the main peaks were proposed and deconvolved after baseline correction, then the area of each peak was calculated using integration method after position alignment, and these operations were performed by Chroma TOF 4.3X software package (Leco Corp., St. Joseph, MI). LECO-Fiehn Rtx5 database was used to annotate the detected substances. The principle of annotation was peak matching and retention time of mass spectrum, and the principle of removal of excess substances was peak detection rate lower than 50% or relative standard deviation greater than 30% in QC samples. The original data of root exudates contained more than 590 substances, which were further screened and standardized. In the screening of original data, the deviation value of a single peak was firstly removed based on the interquartile distance, and the substances with null values less than 50% in the group or null values less than 50% in all samples were retained, the missing values were supplemented by one-half of the minimum value. After that, internal standard normalization method was used to standardize the original data to obtain the relative abundance of substances.

**REFERENCES**

1. Ovreås L, Forney L, Daae FL, Torsvik V. Distribution of bacterioplankton in meromictic Lake Saelenvannet, as determined by denaturing gradient gel electrophoresis of PCR-amplified gene fragments coding for 16S rRNA. Appl Environ Microb. 1997;63:3367.

2. Gardes M, Bruns TD. ITS primers with enhanced specificity for basidiomycetes--application to the identification of mycorrhizae and rusts. Mol Ecol. 1993;2:113-118.

3. Dumonceaux TJ, Hill JE, Briggs SA, Amoako KK, Hemmingsen SM, Van Kessel AG. Enumeration of specific bacterial populations in complex intestinal communities using quantitative PCR based on the chaperonin-60 target. J Microbiol Meth. 2006;64:46-62.

4. Muyzer G, Waal ECD, Uitterlinden AG. Profiling of complex microbial populations by denaturing gradient gel electrophoresis analysis of polymerase chain reaction-amplified genes coding for 16S rRNA. Appl Environ Microb. 1993;59:695-700

5. White TJ, Bruns TD, Lee SB, Taylor JW. Analysis of phylogenetic relationships by amplification and direct sequencing of ribosomal RNA genes. PCR Protocols: A Guide to Methods and Applications. 1990;38:315-322.
